# Systemic Protein Delivery via Inhalable Liposomes: Formulation and Pharmacokinetics

**DOI:** 10.3390/pharmaceutics15071951

**Published:** 2023-07-14

**Authors:** Pranav Ponkshe, Yingzhe Wang, Chalet Tan

**Affiliations:** 1 Department of Pharmaceutics and Drug Delivery, University of Mississippi, Oxford, MS 38677, USA; 2 Preclinical Pharmacokinetic Shared Resource, St. Jude Children’s Research Hospital, Memphis, TN 38105, USA; 3 Department of Pharmaceutical Sciences, University of Tennessee Health Science Center, Memphis, TN 38163, USA

**Keywords:** inhalable liposome, DMPC, systemic protein delivery, pharmacokinetics

## Abstract

The enormous and thin alveolar epithelium is an attractive site for systemic protein delivery. Considering the excellent biocompatibility of phospholipids with endogenous pulmonary surfactant, we engineered dimyristoylphosphatidylcholine (DMPC)-based liposomes for pulmonary administration, using Cy5.5-labeled bovine serum albumin (BSA-Cy5.5) as a model protein payload. The level of cholesterol (Chol) and surface modification with PEG in inhalable liposomes were optimized iteratively based on the encapsulation efficiency, the release kinetics in the simulated lung fluid, and the uptake in murine RAW 264.7 macrophages. The plasma pharmacokinetics of BSA-Cy5.5-encapsulated liposomes with the composition of DMPC/Chol/PEG at 85:10:5 (molar ratio) was studied in mice following intratracheal aerosolization, in comparison with that of free BSA-Cy5.5 solution. The biodisposition of BSA-Cy5.5 was continuously monitored using whole-body near-infrared (NIR) fluorescence imaging for 10 days. We found that the systemic bioavailability of BSA-Cy5.5 from inhaled liposomes was 22%, which was notably higher than that of inhaled free BSA-Cy5.5. The mean residence time of BSA-Cy5.5 was markedly prolonged in mice administered intratracheally with liposomal BSA-Cy5.5, which is in agreement with the NIR imaging results. Our work demonstrates the great promise of inhalable DMPC-based liposomes to achieve non-invasive systemic protein delivery.

## 1. Introduction

Inhalation therapy with small-molecule drugs is a common modality in the clinic to treat various lung conditions, such as asthma, respiratory infections, and chronic obstructive pulmonary diseases [1,2,3]. By directly applying aerosolized medications to the diseased lungs, the primary advantage of inhalation therapy is to instantly achieve high drug levels at the site of action that results in the quick onset of pharmacological effects. The recent development of innovative inhaler devices has made it possible to deliver larger drug doses to the airways with greater precision, efficiency, and patient compliance [4].

Apart from topical pulmonary delivery, the enormous and thin alveolar epithelium in close vicinity of the dense capillary network is an attractive site for systemic drug delivery [5,6]. There are approximately 200–600 million alveoli in lungs, which amount to a total surface area of 70–100 m^2^ in adults. The monolayer of the alveolar epithelium consists of alveolar type I and type II epithelial cells, with tight intercellular junctions. With broad and flattened cytoplasm, alveolar type I cells make up approximately 90% of the alveolar surface area, with a thickness of 0.2–0.7 µm, providing a structural lining with very short diffusion distances for gas exchange between the alveolar space and the capillary blood perfusion (~5 L/min). The alveolar surface is lined with 0.07–0.3 µm thick pulmonary surfactant produced by alveolar type II cells, which is composed of 80% phospholipids (roughly half of them being dipalmitoylphosphatidylcholine (DPPC)), 5–10% neutral lipids (mainly cholesterol), and 5–10% surfactant proteins [6,7].

Therapeutic peptides and proteins constitute the largest group of biological drugs (also known as biologics) that play increasingly important roles in modern medicine owing to their high potency and exquisite target specificity [8,9]. Not orally bioavailable because of their negligible absorption through the intestinal epithelium and rapid metabolic degradation in the gastrointestinal tract, therapeutic peptides and proteins are dosed as ‘injectables’ via parenteral injection. The pulmonary route has long been attempted as a non-invasive ‘needle-free’ alternative to parenteral administration of protein therapeutics [10,11]. The transport of peptides and proteins through the alveolar epithelium depends on the molecular size: peptides and small proteins (<40 KDa) can readily permeate and enter the systemic circulation; the absorption occurs rapidly for peptides (within minutes) but very slowly for large proteins (hours or days) [12]. Apart from this anatomic barrier, there are two clearance mechanisms in alveoli that compete with the systemic absorption of peptides and proteins. First, proteases and peptidases secreted by the alveolar epithelium in the alveolar space—although much less than the gastrointestinal tract—can hydrolyze peptides and small proteins and compromise their bioactivity [12]. Second, abundantly present in the alveolar epithelial lining fluid, alveolar macrophages rapidly endocytose large proteins and remove them from the alveolar epithelium surface [13]. Successful systemic delivery of inhaled peptides/proteins, therefore, necessitates robust formulation strategies that can overcome these biological barriers. Currently, inhaled insulin (5.7 KDa) is the only FDA-approved protein drug that acts systemically, which is formulated with proprietary microsphere technology [14].

Liposomes are phospholipid-based vesicles that have been extensively developed as drug carriers. Currently, there are 18 liposomal drugs approved by the U.S. FDA for the treatment of cancers and other diseases [15]. While the majority of them are indicated for the intravenous route, Arikayce^®^ (liposomal amikacin) is administered as inhalable liposomes [16]. In this study, we explored dimyristoylphosphatidylcholine (DMPC)-based liposomes as a potential platform for the systemic delivery of inhaled proteins. Employing Cy5.5-labeled bovine serum albumin (BSA-Cy5.5) as a model protein payload, a series of BSA-Cy5.5-loaded DMPC-based liposomes with varying levels of cholesterol and PEG were examined in terms of the encapsulation efficiency, release kinetics, and macrophage uptake. The pharmacokinetics of liposomal versus free BSA-Cy5.5 was investigated following intratracheal aerosolization in mice, and whole-body near-infrared (NIR) fluorescence imaging was preformed to evaluate the biodisposition of inhaled BSA-Cy5.5 longitudinally over a 10-day period.

## 2. Materials and Methods

### 2.1. Chemicals

Cy5.5 NHS ester was acquired from Lumiprobe (Hunt Valley, MD, USA). Bovine serum albumin (BSA) was procured from Amresco (Solon, OH, USA). Cholesterol, 1,2-dimyristoyl-sn-glycero-3-phosphocholine (DMPC), and 1,2-distearoyl-*sn*-glycero-3-phosphoethanolamine-N-[amino(polyethylene glycol)-2000] (DSPE-PEG2000) were purchased from Avanti Polar Lipids (Alabaster, AL, USA).

### 2.2. Cell Culture

Murine RAW 264.7 macrophages (ATCC, Manassas, VA, USA) were cultured in high-glucose DMEM medium (Gibco, Waltham, MA, USA) supplemented with glutamine, penicillin (100 U/mL), streptomycin (100 µg/mL), and 10% heat-inactivated fetal bovine serum. Cells were cultured in a 5% CO_2_ humidified incubator at 37 °C.

### 2.3. Conjugation of BSA-Cy5.5

Cy5.5 NHS ester (8.66 mg, dissolved in 100 µL of DMSO) was added drop-wise to BSA (50 mg) dissolved in 1 mL of sodium bicarbonate (0.1 M, pH 8.5) under constant stirring. The conjugation mixture was stirred at room temperature for 1 h, then subsequently at 4 °C overnight. The mixture was then diluted 30-times in sterile PBS (pH 7.4) and passed through 0.2-µm nylon membrane syringe filter to remove the unreacted dye. BSA-Cy 5.5 was concentrated using Vivaspin centrifugal filters (10 KDa MWCO, Sartorius, Göttingen, Germany) under centrifugation at 10,000× *g* for 5 min. The resulting BSA-Cy5.5 was reconstituted in 500 µL of PBS (pH 7.4, with 0.025% sodium azide) to obtain a final concentration of 100 mg/mL and stored in −20 °C.

To establish a standard curve for the quantification of BSA-Cy5.5, a series of BSA-Cy5.5 solutions in a range of 0.1–10 µg/mL were prepared in PBS, which were measured in black-walled 96-well plate for fluorescence intensity (λ_excitation_ = 684 nm; λ_emission_ = 700 nm) using an Odyssey CLx system (Li-Cor, Lincoln, NE, USA).

### 2.4. Preparation and Characterization of BSA-Cy.5.5-Encapsulated Liposomes

A well-established thin-film hydration method was used to prepare BSA-Cy5.5-encapsulated liposomes [15]. DMPC, cholesterol, and DSPE-PEG2000 were dissolved in 0.5 mL of ethanol (Sigma, St. Louis, MO, USA) at varying molar ratios (with a total lipid content fixed at 0.3 mg) in a 5 mL round-bottom flask. The mixture was dried using a rotary evaporator to obtain a thin film, which was further dried under vacuum overnight. The dry film was rehydrated with 100 µL of the BSA-Cy5.5 solution (100 mg/mL in PBS, pH 7.4) at 33 °C under vigorous vortexing for 10 min. The resulting multilamellar liposomes were subjected to probe sonication (Branson Sonifier SFX250, Emerson, St. Louis, MO, USA) to yield unilamellar liposomes. Sonication was performed with 40% amplitude and pulse mode (5 s on and 10 s off cycle, repeated 6 times) in an ice bath. The liposomes were kept at 4 °C for 1 h before performing further analysis. BSA-Cy5.5-loaded liposomes were separated from unloaded, free BSA-Cy5.5 by using Vivaspin centrifugal filters (300 KDa MWCO) under centrifugation at 10,000× *g* for 5 min. Owing to the size exclusion, liposomes remained in the top compartment, whereas the unencapsulated, free BSA-Cy5.5 was filtered through the membrane into the bottom compartment.

To determine the BSA-Cy5.5 concentration, BSA-Cy5.5-encapsulated liposomes were diluted with PBS and lysed using 0.1% Triton X-100 at 1:1 ratio (by volume) to burst release the encapsulated BSA-Cy5.5, which was analyzed in black-walled 96-well plates for the fluorescence intensity (λ_excitation_ = 684 nm; λ_emission_ = 700 nm) using an Odyssey CLx system. A standard curve of BSA-Cy5.5 (dissolved in a mixture of PBS and 0.1% Triton X-100 at 1:1 ratio) that correlated the fluorescence intensity with the BSA-Cy5.5 concentration in a range of 0.1–10 µg/mL was established. The encapsulation efficiency (EE) of BSA-Cy5.5 was calculated as the percentage of the loaded versus the input BSA-Cy5.5.

The hydrodynamic diameter and the ξ potential of liposomes were measured using a Malvern Zetasizer Nano ZS (Worcestershire, UK).

### 2.5. In Vitro Release Study

BSA-Cy5.5-loaded liposomes (500 µL) were sealed in a dialysis bag (300 KDa MWCO, Spectra/Por membrane, Repligen, Waltham, MA, USA), which was immersed in 200 mL of the simulated lung fluid (pH 7.4) at 37 °C under constant stirring. The formula for the simulated lung fluid is as follows: 0.095 g/L of magnesium chloride, 6.019 g/L of sodium chloride, 0.298 g/L potassium chloride, 0.126 g/L of disodium hydrogen phosphate, 0.063 g/L of sodium sulfate, 0.368 g/L of calcium chloride dihydrate, 0.574 g/L of sodium acetate, 2.604 g/L of sodium bicarbonate, and 0.097 g/L of sodium citrate dihydrate [17]. To maintain the sink conditions, the simulated lung fluid was refreshed every 2 h. At pre-determined time points, 2–5 µL of samples was withdrawn from the dialysis bag, diluted with 50 µL of PBS, lysed with 0.1% Triton X-100 solution, and analyzed in black-walled 96-well plates for the fluorescence intensity (λ_excitation_ = 684 nm; λ_emission_ = 700 nm) using an Odyssey CLx system. To quantify the release kinetics, the first-order release kinetics equation *C_t_*/*C_o_* = *e^−kt^* was applied, where *C_t_* and *C_o_* were the concentration of BSA-Cy5.5-loaded liposomes at the sample collection time *t* and the initial time zero, respectively. The first-order release rate constant (*k*) was derived from the best-fit nonlinear regression (GraphPad Prism, Boston, MA, USA), which was used to calculate the release half-life (*t*_1/2,*release*_ = 0.693/*k*).

### 2.6. Macrophage Uptake of BSA-Cy5.5-Loaded Liposomes

RAW 264.7 macrophages were seeded in 6-well plates (5 × 10^5^ cells/well in 1 mL of complete DMEM) and incubated overnight. The cells were treated with free BSA-Cy5.5, or BSA-Cy5.5-loaded DMPC-based liposomes with varying levels of PEG (0–10%) for 4 h. The concentrations of BSA-Cy5.5 and the total lipid content of each formulation were kept constant at 0.5 mg/mL and 3 mg/mL, respectively. Following 4 h incubation, the cells were washed twice with PBS (0.5 mL/well), harvested with a cell scraper, and centrifuged at 1000× *g* for 2 min. The cell pellets were reconstituted in 0.5 mL of 0.05% trypsin (with 0.5 mM EDTA) and incubated at 37 °C for 5 min to remove BSA-Cy5.5 adsorbed outside of the cell membrane, washed three times with PBS (0.5 mL/well), and suspended in 200 µL of FACS buffer (2% FBS and 2.5 mM EDTA in HBSS). The mean fluorescence intensity (MFI) of Cy5.5 was measured from 10,000 cells utilizing flow cytometry (Guava easyCyte, Luminex, Austin, TX, USA).

### 2.7. Pharmacokinetics of Liposomal Versus Free BSA-Cy5.5

NIH Swiss mice (8–10 weeks old, 22–25 g) were purchased from Charles River (Wilmington, MA, USA). For intratracheal (IT) administration, the mice (*n* = 4) were anesthetized with intraperitoneal injection of a cocktail of ketamine (87.5 mg/kg) and xylazine (12.5 mg/kg). A microsprayer aerosolizer (PennCentury model IA-1C-M, Bio Jane Trading Ltd, Hong Kong, China) loaded with 2 mg of free BSA-Cy5.5 solution or BSA-Cy5.5-loaded liposomes (30 µL in PBS) was inserted into the mouse trachea and sprayed. The microsprayer was removed immediately, and the mice were kept in an upright position for 1 min to ensure the dose completely entered the deep lungs. For intravenous (IV) administration, 1 mg of free BSA-Cy5.5 solution or BSA-Cy5.5-loaded liposomes (50 µL in PBS) was injected into the tail vein of mice (*n* = 4). At the pre-determined time points, blood samples were collected via tail vein nicking. The plasma samples were diluted in PBS and analyzed for the fluorescence intensity. The BSA-Cy5.5 concentration was determined according to the standard curves described above.

Non-compartmental analysis was performed by using WinNonlin version 6.4 (Certara, Mountain View, CA, USA) to obtain the pharmacokinetic parameters of liposomal versus free BSA-Cy5.5, including the mean resident time in plasma (*MRT*) and the area under the curve (*AUC_0−last_*). The pulmonary bioavailability (*F*) of BSA-Cy5.5 of the respective formulation was calculated using the following equation: *F* = (*AUC_0−last,IT_*/ *AUC_0−last,IV_*) × (*IV dose*/*IT dose*) × 100%

### 2.8. Whole-Body NIR Fluorescence Imaging

The BSA-Cy5.5 level in mice (*n* = 3) was detected using whole-body near-infrared (NIR) imaging (Odyssey CLx) at 2, 6, 18, and 24 h post-dosing and then daily for up to 10 days. The excitation and emission wavelengths were set at 684 nm and 700 nm, respectively. Immediately prior to the NIR imaging, the mice were anesthetized with intraperitoneal injection of a cocktail of ketamine (87.5 mg/kg) and xylazine (12.5 mg/kg). Rectangular areas that covered from the nose to the base of the tail were boxed for scanning. Images were acquired in 7.5 min at 169-μm resolution with a gain of L1. Identical instrumental settings were applied to all mice. Image Studio software (version 2.1.10, Li-Cor) was used to analyze the NIR images.

### 2.9. Statistical Analysis

All data are shown as the mean ± SD (*n* ≥ 3). Statistical analysis was conducted using the Student’s *t*-test for comparison between two groups (GraphPad Prism).

## 3. Results

### 3.1. Sythesis and Characterization of BSA-Cy5.5-Loaded Liposomes

As a model protein, BSA was conjugated to Cy5.5 to allow for sensitive quantification by the NIR fluorescence detection. By utilizing the standard curves that correlate the Cy5.5 concentration with the NIR fluorescence intensity, we determined that the resulting BSA-Cy5.5 had a BSA/Cy5.5 molar ratio of 1:8.

DMPC was chosen as the phospholipid to prepare BSA-Cy5.5-loaded liposomes owing to its low transition temperature at 23 °C. To develop an optimal formulation for BSA-Cy5.5, we prepared a series of liposomes (Table 1) with varying molar ratios of DMPC, cholesterol (Chol), and DSPE-PEG2000 (PEG). While all formulations exhibited comparable hydrodynamic particle size (115–135 nm) and ξ potential (between −6 and −13 mV) with a narrow polydispersity index (PDI, 0.22–0.33), the lowest encapsulation efficiency of BSA-Cy5.5 was observed with DMPC liposomes without the inclusion of Chol, which was notably lower than those of the formulations with 10% or 20% Chol, irrespective of the PEG level. However, further increasing Chol to 30% had an adverse effect on the encapsulation efficiency (EE) of BSA-Cy5.5.

The stability of BSA-Cy5.5-encapsulated liposomes with the composition of DMPC/Chol/PEG at 85:10:5 (molar ratio) was evaluated following 6-day storage at 4 °C, in comparison with the liposomes made of pure DMPC. Minimal change was observed in the former liposomes in terms of the particle size, PDI, and the encapsulated BSA-Cy5.5 level. By contrast, the pure DMPC liposomes were only stable for the initial 4 days. By day 6, the particle size became enlarged (179.5 ± 10.7 nm) with higher PDI (0.43), while the encapsulated BSA-Cy5.5 was reduced by nearly 30%, indicating the loss of structural integrity of the pure DMPC liposomes.

### 3.2. Factors Influencing the Release Kinetics of BSA-Cy5.5 from DMPC-Based Liposomes

**Figure 1:**
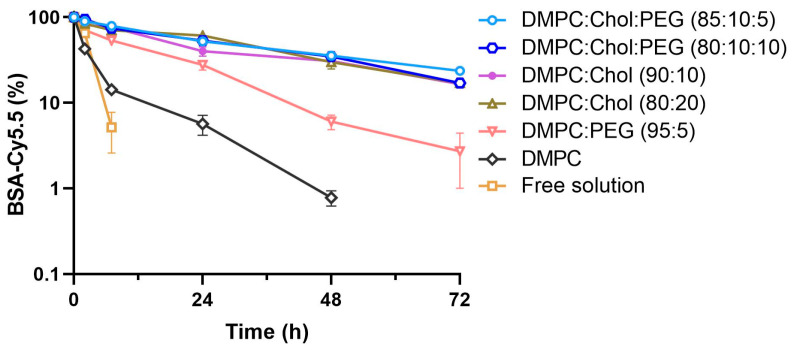
Release kinetics of BSA-Cy5.5 from DMPC-based liposomes. BSA-Cy5.5-encapsulated DMPC-based liposomes were sealed in a dialysis bag (300 KDa MWCO), which was immersed in the simulated lung fluid at 37 °C under the sink conditions. At pre-determined time points, samples were withdrawn from the dialysis bag and were analyzed for the BSA-Cy5.5 concentration based on the fluorescence intensity. Results are shown as the mean ± SD (*n* = 3) and are representative of three independent experiments.

Next, we examined the release profiles of BSA-Cy5.5 from liposomes with varying levels of DMPC, Chol, and PEG. BSA-Cy5.5-encapsulated DMPC-based liposomes were sealed in a dialysis bag (300 KDa MWCO), which was immersed in the simulated lung fluid under the sink conditions. The release kinetics of BSA-Cy5.5 was examined by monitoring its concentration inside the dialysis bag. For BSA-Cy5.5 molecules to be released into the sink, they first need to be liberated from liposomes and subsequently diffuse through the dialysis membrane into the release sink. To ascertain that the dialysis membrane is not a rate-limiting barrier, the diffusion of free BSA-Cy5.5 was studied as a control, which showed a release half-life (*t*_1/2,*release*_) of 0.4 h. The encapsulation of BSA-Cy5.5 into liposomes significantly slowed down the release kinetics (Figure 1) and prolonged *t*_1/2,*release*_, depending on the lipid compositions (Table 2), indicating that the liberation of BSA-Cy5.5 from liposomes is the rate-limiting step during the release process. The pure DMPC liposomes without the inclusion of Chol and PEG exhibited the quickest release kinetics (*t*_1/2,*release*_ = 2.4 h), whereas 5% PEG DMPC liposomes (without Chol) extended *t*_1/2,*release*_ to 9.5 h, likely due to the steric stabilizing effect conferred by the PEG corona. On the other hand, the incorporation of 10% or 20% Chol markedly prolonged *t*_1/2,*release*_ to over 24 h, reflecting the improved DMPC membrane stability and less leakage of the protein payload. The effect of 5% or 10% PEG on the release kinetics of DMPC/Chol liposomes was negligible.

### 3.3. PEG Modification of Liposomes Attenuates the Macrophage Uptake of Liposomal BSA-Cy5.5

**Figure 2:**
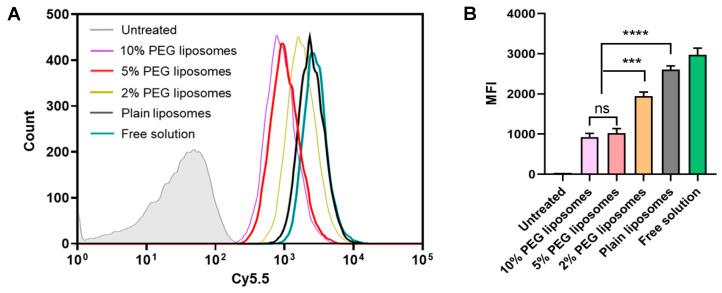
PEG surface modification attenuates the macrophage uptake of BSA-Cy5.5-encapsulated DMPC-based liposomes. RAW 264.7 macrophages were treated with free BSA-Cy5.5 or BSA-Cy5.5-loaded DMPC-based liposomes with varying levels of PEG (0–10%) for 4 h. The concentration of BSA-Cy5.5 was kept constant at 0.5 mg/mL for all formulations. RAW 264.7 macrophages were harvested, washed, and analyzed via flow cytometry. (**A**) Representative flow cytometry histograms of Cy5.5 fluorescence in RAW 264.7 macrophages. (**B**) The mean fluorescence intensity (MFI) of Cy5.5 in RAW 264.7 macrophages. Results are shown as the mean ± SD (*n* = 3) and are representative of three independent experiments. Statistically significant difference between the groups was evaluated using unpaired two-tailed Student’s *t*-test (*** *p* < 0.001, **** *p* < 0.0001).

A critical barrier for inhaled liposomes to overcome before delivering the protein payload into the systemic circulation is the clearance by alveolar macrophages. As PEGylated liposomes can become ‘stealth’ to phagocytes, we examined the effect of modifying the liposomal surface with PEG on the macrophage uptake of BSA-Cy5.5-encapsulated DMPC-based liposomes. To search for an optimal PEG level, a series of DMPC-based liposomes with Chol set at 10% and PEG ranging from 0 to 10% were prepared, and the uptake by murine RAW 267.4 macrophages was evaluated via flow cytometry analysis. To quench the fluorescence signal outside of the cell membrane following the treatment with free or liposomal BSA-Cy5.5 for 4 h, RAW 267.4 macrophages were incubated with trypsin/EDTA solution and were washed thoroughly. We found that without PEG, the plain DMPC liposomes were avidly taken up by RAW 267.4 macrophages, to an extent comparable to that of free BSA-Cy5.5. As the PEG level was increased from 2% to 5 or 10%, there was a significant reduction in the fluorescence intensity (*p* < 0.001), indicating that PEG modification of DMPC-based liposomes attenuates the macrophage uptake (Figure 2). Since no significant difference was observed between 5% and 10% PEG, we chose 5% PEG liposomes (DMPC/Chol/PEG = 85:10:5 molar ratio) for subsequent in vivo studies.

### 3.4. Inhaled DMPC-Based Liposomes Increase the Systemic Absorption of BSA-Cy5.5 in Mice

**Figure 3:**
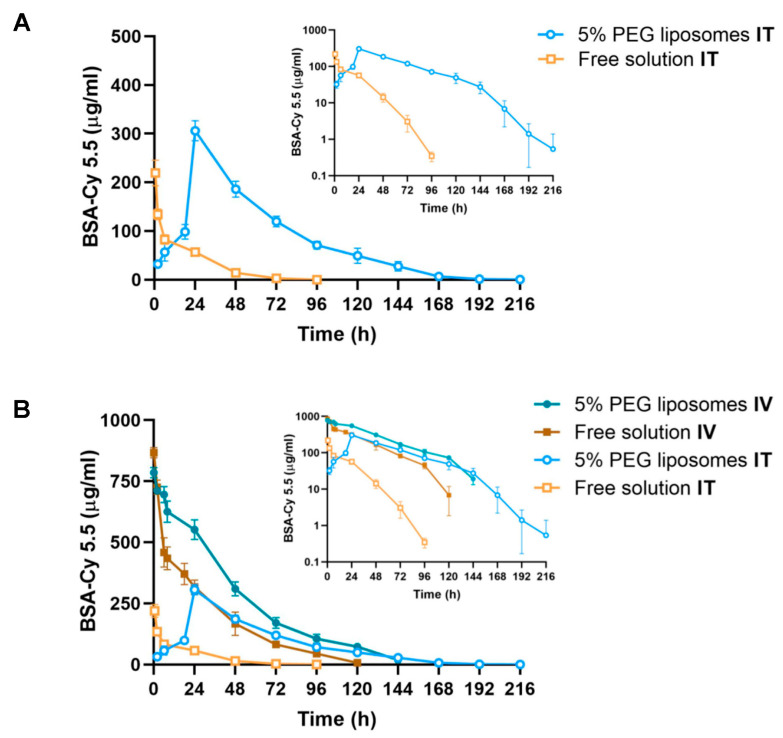
The plasma BSA-Cy5.5 level versus time profiles in mice following IT and IV administration of BSA-Cy5.5-loaded DMPC-based liposomes in comparison with free BSA-Cy5.5 solution. (**A**) The mice were dosed with liposomal or free BSA-Cy5.5 (2 mg) via IT aerosolization. (**B**) The mice were dosed with liposomal or free BSA-Cy5.5 (1 mg) via IV injection. The inserted plots are shown in the semi-log scale. The plasma BSA-Cy5.5 level was determined via fluorescence intensity. Results are represented as the mean ± SD (*n* = 4).

To investigate the impact of inhaled DMPC-based liposomes on the systemic absorption of BSA-Cy5.5, we carried out pharmacokinetic studies in mice following IT aerosolization (2 mg of BSA-Cy5.5). As shown in Figure 3A, BSA-Cy5.5 was detected in plasma as early as 2 h post-IT dosing of 2 mg of BSA-Cy5.5-loaded liposomes (DMPC/Chol/PEG = 85:10:5 molar ratio), which reached the maximum plasma level (*C_max_* = 306 ± 21 µg/mL) at 24 h followed by a protracted decline and remained detectable until 216 h. By contrast, free BSA-Cy5.5 was absorbed immediately into the bloodstream following IT aerosolization, and the plasma BSA-Cy5.5 level peaked at 0.5 h (*C_max_* = 219 ± 26 µg/mL), which dropped rapidly and became undetectable after 96 h. The area under the plasma concentration curve (*AUC_0-last,IT_*) of liposomal BSA-Cy5.5 was 5.7-fold that of free BSA-Cy5.5 solution. As the elimination phase of the plasma BSA-Cy5.5 concentration versus time curves did not exhibit simple monoexponetial decline (Figure 3A insert), the elimination half-life of BSA-Cy5.5 could not be properly estimated. Instead, the mean residence time (MRT)—a parameter that measures the average time that the administered molecules remain in the circulation—was calculated. We found that the MRT of liposomal versus free BSA-Cy5.5 was 58.6 ± 4.4 h and 19.3 ± 1.6 h, respectively. These results indicate that inhaled DMPC-based liposomes markedly increase the systemic absorption of BSA-Cy5.5 and prolong its circulation time.

Furthermore, in order to determine the systemic bioavailability of IT-aerosolized BSA-Cy5.5, we also studied the pharmacokinetics of liposomal versus free BSA-Cy5.5 following IV injection (1 mg of BSA-Cy5.5). Although *C_max_* of BSA-Cy5.5 in plasma was comparable for both formulations, the plasma BSA-Cy5.5 level declined more gradually with longer MRT (39.2 ± 1.3 h) in mice receiving liposomal BSA-Cy5.5 compared to that of free BSA-Cy5.5 solution (MRT = 30.3 ± 1.6 h), reflecting the slower elimination of BSA-Cy5.5 owing to the protection from liposomal encapsulation (Figure 3B). After the dose normalization, the ratio of *C_max,IT_*/*C_max,IV_* for liposomal and free BSA-Cy5.5 was 0.19 and 0.11, respectively. The pulmonary bioavailability of liposomal and free BSA-Cy5.5 was 22.2 ± 1.8% and 6.9 ± 0.8%, respectively. It is worth noting that all four plasma BSA-Cy5.5 concentration versus time curves showed steeper terminal slopes as the plasma concentrations fell below 30–100 µg/mL (Figure 3B insert), suggesting the nonlinear pharmacokinetics and saturable clearance of BSA-Cy5.5 at a higher plasma concentration. The bioavailability of BSA-Cy5.5 may, therefore, be dose-dependent. The pharmacokinetic parameters are summarized in Table 3.

**Figure 4:**
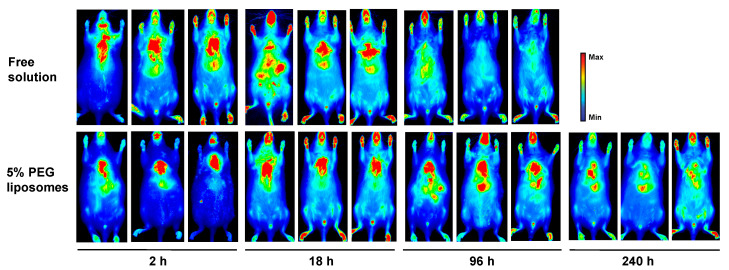
Whole-body NIR imaging of mice following IT administration of BSA-Cy5.5-loaded DMPC-based liposomes in comparison with free BSA-Cy5.5 solution. Mice were treated with liposomal or free BSA-Cy5.5 (2 mg) via IT aerosolization. The BSA-Cy5.5 level of in mice was detected using whole-body NIR imaging at 2–240 h post-IT dosing. Identical instrumental settings were applied to all mice (*n* = 3).

The biodisposition of inhaled liposomal versus free BSA-Cy5.5 was assessed longitudinal using whole-body NIR fluorescence imaging. As shown in Figure 4, the fluorescence signal was most localized in the lungs at 2 h post-IT aerosolization of both formulations, demonstrating the dispersion of intratracheally administered aerosols into the deep lungs. In the mice receiving free BSA-Cy5.5 solution, the fluorescence intensity in the lungs was visibly reduced at 18 h, a clear indication of the removal of BSA-Cy5.5 from the lungs. The NIR images also revealed that the fluorescence signals were distributed to the liver and other organs over time, which faded into the background at 96 h, consistent with the findings that the plasma BSA-Cy5.5 level fell below the detection limit after 96 h (Figure 3A). By contrast, in mice receiving liposomal BSA-Cy5.5, the fluorescence intensity in the lungs remained high at 18 h, which gradually declined and distributed to the other organs over the next 10 days. These images provide strong evidence that inhaled DMPC-based liposomes greatly extend the retention of BSA-Cy5.5 in the deep lungs.

## 4. Discussion

Liposomes are versatile drug carriers that are tailored by the choices of phospholipids, lamellarity, and surface properties [18]. In this study, we aimed to develop unilamellar liposomes that can be broadly applicable for systemic protein delivery following the inhalation route. DPPC is most commonly used in inhalable liposomes, because it is the dominant phospholipid in the pulmonary surfactant produced and recycled by alveolar type II epithelial cells [19]. However, with a phase transition temperature of 41 °C, the preparation of DPPC liposomes requires heating conditions above 50 °C [20,21], which may inadvertently cause stability issues for heat-labile macromolecules. Instead, we chose DMPC, which has a phase transition temperature of 23 °C, to engineer protein-encapsulated liposomes at 33 °C.

Compared to phospholipids with longer aliphatic chains, the lipid bilayer formed by DMPC alone is in a more fluid liquid-crystalline phase. A critical component in the liposomal composition is cholesterol, a neutral lipid that controls the membrane stoutness and rigidity [22]. The important role of cholesterol in DMPC-based liposomes is evident from the profiles of encapsulation and release of BSA-Cy5.5. Incorporation of 10 or 20% cholesterol in DMPC-based liposomes not only improved the encapsulation efficiency for BSA-Cy5.5 but also better retained and released the payload in a sustained manner. However, excessive cholesterol compromised the performance of liposomes, which is in agreement with the literature [23].

An important clearance mechanism in the alveolar space that competes with systemic protein absorption is posed by alveolar macrophages. Large proteins, such as albumin and IgG, are subjected to rapid clearance by alveolar macrophages [13]. Depending on the size and the surface modifications, liposomes may also be recognized and taken up by alveolar macrophages [24]. As PEGylated nanoparticles are known to shield the surface from aggregation, opsonization, and recognition by the mononuclear phagocytic system owing to the steric hindrance [25], we hypothesized that PEG modification of DMPC-based liposomes could deter macrophage uptake. Indeed, we observed avid macrophage uptake of free BSA-Cy5.5 as well as the plain DMPC liposomes carrying BSA-Cy5.5, which was pronouncedly reduced by 5–10% PEG liposomes, supporting the surface modification of inhalable liposomes with PEG for systemic protein delivery. It is worth noting that PEGylation of therapeutic proteins can also increase the local residence time as well as the drug exposure in the lungs [26,27].

The most interesting revelation from this study is that the pulmonary bioavailability of BSA-Cy5.5 was markedly increased by inhaled DMPC-based liposomes (DMPC/Chol/PEG = 85:10:5 molar ratio). The transport and pulmonary absorption of albumin has been well investigated previously. As a large protein with a molecular weight of 66.5 KDa, the permeation of albumin across the alveolar epithelium is mediated by receptor-mediated transcytosis instead of passive diffusion [28]. Following the intratracheal instillation, the bioavailability of albumin was 4.5% in rats with a *t_max_* of 24 h [12]. In this study, we observed the immediate absorption of BSA-Cy5.5 following the intratracheal aerosolization of free BSA-Cy5.5 solution, likely attributable to the instantaneous dispersion of aerosols into the deep lungs. However, the absorption phase was short-lived, suggesting rapid removal of BSA-Cy5.5 from the alveolar surface, as albumin undergoes rapid clearance by alveolar macrophages [29]. By striking contrast, the inhalation of BSA-Cy5.5-encapsulated DMPC-based liposomes resulted in a protracted absorption phase, accompanied by much higher *C_max_* and *AUC* of BSA-Cy5.5 in plasma. There are two plausible causes responsible for the enhanced systemic bioavailability of BSA-Cy5.5. First, the retention of BSA-Cy5.5 in the alveolar space was greatly extended owing to the encapsulation in DMPC-based liposomes, allowing for prolonged protein transport through the alveolar epithelium into the systemic circulation. Second, PEGylated liposomes could shield BSA-Cy5.5 from alveolar macrophages and release the payload in a sustained manner.

## 5. Conclusions

By optimizing the levels of cholesterol and PEG, DMPC-based liposomes can be tuned to increase the systemic absorption of protein payload following inhaled delivery to the deep lungs. Our work demonstrates great promise for inhalable DMPC-based liposomes to achieve non-invasive systemic protein delivery.

## Figures and Tables

**Table 1:** Physicochemical characterization of BSA-Cy5.5-encapsulated DMPC-based liposomes.

Compositions (Molar Ratio)	EE (%)	Particle Size (nm)	PDI	ξ Potential (mV)
DMPC	52.1 ± 2.3	127.1 ± 3.9	0.23 ± 0.03	−12.7 ± 2.1
DMPC/PEG (95:5)	50.6 ± 1.5	115.6 ± 2.6	0.29 ± 0.01	−9.5 ± 0.7
DMPC/Chol (90:10)	69.6 ± 1.4	127.1 ± 5.5	0.28 ± 0.06	−11.8 ± 0.3
DMPC/Chol (80:20)	67.3 ± 2.6	132.2 ± 2.5	0.24 ± 0.03	−6.3 ± 2.7
DMPC/Chol/PEG (85:10:5)	70.4 ± 0.7	129.1 ± 2.8	0.22 ± 0.04	−7.7 ± 0.5
DMPC/Chol/PEG (80:10:10)	71.2 ± 2.0	125.5 ± 0.6	0.33 ± 0.02	−11.1 ± 1.5
DMPC/Chol/PEG (60:30:10)	55.7 ± 3.1	132.1 ± 2.7	0.32 ± 0.01	−12.0 ± 1.0

**Table 2:** The release kinetics of BSA-Cy5.5 from DMPC-based liposomes.

Compositions (Molar Ratio)	Release Rate Constant (h^−1^)	Release Half-Life (h)	Goodness of Fit
Free solution	1.850 ± 0.235	0.4 ± 0.1	0.994
DMPC	0.291 ± 0.060	2.4 ± 0.5	0.972
DMPC/PEG (95:5)	0.073 ± 0.023	9.5 ± 3.9	0.977
DMPC/Chol (90:10)	0.029 ± 0.004	24.3 ± 4.4	0.969
DMPC/Chol (80:20)	0.026 ± 0.003	26.2 ± 2.8	0.991
DMPC/Chol/PEG (85:10:5)	0.028 ± 0.002	25.1 ± 2.3	0.995
DMPC/Chol/PEG (80:10:10)	0.025 ± 0.003	27.4 ± 2.7	0.990

**Table 3:** The pharmacokinetic parameters for BSA-Cy5.5-loaded DMPC-based liposomes versus free BSA-Cy5.5 solution following IT and IV administration in mice.

Pharmacokinetic Parameters	5% PEG Liposomes		Free Solution	
	IT	IV	IT	IV
*Dose* (mg)	2	1	2	1
*C_max_* (µg/mL)	306 ± 21	785 ± 27	219 ± 26	868 ± 20
*t_max_* (h)	24	0.08	0.5	0.08
*AUC_0-last_* (µg/mL·h)	16,761 ± 1333	37,765 ± 2915	2927 ± 340	21,299 ± 1972
*F* (%)	22.2 ± 1.8	100 ± 7.7	6.9± 0.8	100 ± 9.3
*MRT* (h)	58.6 ± 4.4	39.2 ± 1.3	19.3 ± 1.6	30.3 ± 1.1

## Data Availability

Not applicable.
